# Novel Mg-Doped SrMoO_3_ Perovskites Designed as Anode Materials for Solid Oxide Fuel Cells

**DOI:** 10.3390/ma9070588

**Published:** 2016-07-19

**Authors:** Vanessa Cascos, José Antonio Alonso, María Teresa Fernández-Díaz

**Affiliations:** 1 Instituto de Ciencia de Materiales de Madrid, CSIC, Cantoblanco, 28049 Madrid, Spain; jaalonso@icmm.csic.es; 2 Institut Laue Langevin, BP 156X, Grenoble 38042, France; ferndiaz@ill.fr

**Keywords:** anode, IT-SOFC, SrMoO_3_, perovskite, neutron diffraction

## Abstract

SrMo_1−x_M_x_O_3−δ_ (M = Fe and Cr, x = 0.1 and 0.2) oxides have been recently described as excellent anode materials for solid oxide fuel cells at intermediate temperatures (IT-SOFC) with LSGM as the electrolyte. In this work, we have improved their properties by doping with aliovalent Mg ions at the B-site of the parent SrMoO_3_ perovskite. SrMo_1−x_Mg_x_O_3−__δ_ (x = 0.1, 0.2) oxides have been prepared, characterized and tested as anode materials in single solid-oxide fuel cells, yielding output powers near 900 mW/cm^−2^ at 850 °C using pure H_2_ as fuel. We have studied its crystal structure with an “in situ” neutron power diffraction (NPD) experiment at temperatures as high as 800 °C, emulating the working conditions of an SOFC. Adequately high oxygen deficiencies, observed by NPD, together with elevated disk-shaped anisotropic displacement factors suggest a high ionic conductivity at the working temperatures. Furthermore, thermal expansion measurements, chemical compatibility with the LSGM electrolyte, electronic conductivity and reversibility upon cycling in oxidizing-reducing atmospheres have been carried out to find out the correlation between the excellent performance as an anode and the structural features.

## 1. Introduction

Solid oxide fuel cells at intermediate temperatures (IT-SOFC) are electrochemical devices able to convert the energy involved in the combustion of a fuel directly into electrical energy. IT-SOFCs work at intermediate temperatures, typically between 700 °C and 850 °C; therefore, the reaction kinetics is extremely favored, and the efficiency of the energy conversion process is very high, compared to other low-temperature fuel cells. The fuel oxidation reaction in SOFC happens in the anode. SOFCs often use anodes based on Ni-YSZ (yttria-stabilized zirconia) and Ni-LDC (lanthanum-dope ceria) cermets. These composite anodes have an excellent catalytic activity for the fuel-oxidation reaction and high electronic and ionic conductivity, but unfortunately, these materials promote carbon formation during the direct oxidation of hydrocarbon fuels and suffer from sintering problems during the cell operation [1,2,3]. Furthermore, Ni-based anodes have the disadvantage of being contaminated with H_2_S traces contained in H_2_ [4].

In order to avoid the problems associated with the cermet-based anodes, single-phase active materials have been investigated with the ABO_3_ perovskite structure. By suitably choosing stable oxide compounds in reducing atmospheres, these materials can provide enough electronic and ionic conductivity to perform as anodes in IT-SOFC. The SrMoO_3_ cubic perovskite with Mo^4+^ at the octahedral B positions has an extremely high electrical conductivity at room temperature (10^4^ S∙cm^−1^ [5]); moreover, molybdenum is a very suitable element to catalyze the fuel-oxidation reaction. Unfortunately, this oxygen-stoichiometric oxide cannot exhibit the required oxygen-ion diffusion and conductivity. In previous works, the Mo ions were partially replaced by 10% and 20% aliovalent elements, namely Fe^3+^ and Cr^3+^ [6,7], thus inducing the creation of oxygen vacancies in the perovskite material. We demonstrated that Fe and Cr doping promotes the ionic conductivity of these oxides, thus combining excellent mixed ionic and electronic conduction (MIEC) properties that make them excellent anode materials.

Following the same strategy, taking advantage of the excellent metallic conduction properties of SrMoO_3_, in the present work, we show that doping with aliovalent Mg^2+^ ions at the B-site is also extremely effective for the mentioned purpose. Mg^2+^ ions were chosen because they are able to adopt an octahedral coordination in a perovskite structure, and the large ionic size (0.72 Å) [8] may lead to an expansion of the unit-cell dimensions, thus promoting the ionic diffusion across the solid. Additionally, avoiding the use of transition metals (like Fe^3+^ and Cr^3+^) in the anode could prevent the diffusion across the electrolyte and hinder the induction of electronic conductivity. Moreover, the use of Mg^2+^ is perfectly compatible with the electrolyte LSGM, also containing this element. In the present case, 10% and 20% Mg^2+^ were introduced in the perovskite, developing new mixed conductors with potential application as anodes in SOFCs at intermediate temperature.

SrMo_1−x_Mg_x_O_3−δ_ (x = 0.1 and 0.2) materials have been prepared and characterized by different techniques, and finally, their performance was evaluated as anodes in a test cell, using SrCo_0.8_Fe_0.2_O_3−δ_ (SCFO) as the cathode and LSGM as the electrolyte. The structural characterization was carried out from an in situ temperature-dependent neutron powder diffraction (NPD) study in the 25–800 °C range, under the actual working conditions of a SOFC. Additionally, thermal expansion, chemical compatibility, electrical conductivity and the reversibility of the oxidation-reduction process were also investigated.

## 2. Experimental Section

SrMo_1__−__x_Mg_x_O_3−δ_ (x = 0.1, 0.2) polycrystalline samples were synthesized by soft-chemistry procedures. Stoichiometric amounts of Sr(NO_3_)_2_, (NH_4_)_6_Mo_7_O_24_·4H_2_O and Mg(NO_3_)_2_·6H_2_O were dissolved in a 10% citric acid solution (50 g of citric acid dissolved in 500 mL of water). After removing the solvent by gentle heating, the formed organic resins were decomposed at 600 °C for 12 h in air. Oxidized scheelite phases of composition SrMo_1−x_Mg_x_O_4−δ_, containing Mo^6+^ ions, were identified by XRD after the treatment at 600 °C in air. A final treatment at 1050 °C in a tubular furnace under a H_2_ (5%)/N_2_ flow for 15 h led to the formation of the reduced perovskite oxide. 

The initial characterization of the product was carried out by X-ray diffraction (XRD) with a Bruker D8 Advanced diffractometer (40 kV, 30 mA), controlled by DIFFRACT^PLUS^ software, in Bragg–Brentano configuration with CuK_α_ radiation (λ = 1.5418 Å ) and a PSD (position-sensitive detector). A filter of nickel allows the complete removal of CuK_β_ radiation. The data were obtained between 10° and 64° in steps of 0.02°. 

NPD data were collected in the diffractometer D2B at the Institut Laue-Langevin, (Grenoble, France), with a neutron wavelength λ = 1.594 Å within the angular 2θ range from 10°–160° for x = 0.1, and at the HRPT diffractometer of the SINQ spallation source (PSI, Villigen, Switzerland), with λ = 1.494 Å within the 2θ range from 10°–164° for x = 0.2. About 2 g of the samples were contained in vanadium cans and studied at 25 °C. For the temperature-dependent study, a selected sample contained in a vanadium cylinder was placed in the isothermal zone of a furnace with a vanadium resistor operating under vacuum (PO_2_ ≈ 10^−^^6^ Torr) coupled to the D2B diffractometer. The measurements were carried out at 25, 200, 400, 600 and 800 °C for x = 0.1. In all cases, the collection times were 2 h per pattern. The diffraction data were analyzed by the Rietveld method [9] with the FULLPROF program [10] and the use of its internal tables for scattering lengths. The line shape of the diffraction peaks was generated by a pseudo-Voigt function. In the final run, the following parameters were refined: scale factor, background coefficients, zero-point error, pseudo-Voigt corrected for asymmetry parameters and positional coordinates. Isotropic thermal factors for all of the metal atoms and the anisotropic ones for oxygen atoms were also refined for the NPD data. The coherent scattering lengths for Sr, Mo, Mg and O were 7.02, 6.715, 5.375 and 5.805 fm, respectively.

Thermal analysis was carried out in a Mettler TA3000 system equipped with a TC15 processor unit. Thermogravimetric (TG) curves were obtained in a TG50 unit, working at a heating rate of 10 °C∙min^−^^1^, in an O_2_ flow of 100 mL·min^−^^1^ from 35–900 °C using about 50 mg of sample in each experiment.

Measurements of the thermal expansion coefficient and electrical conductivity required the use of sintered samples. For this purpose, pellets of SrMo_1__−__x_Mg_x_O_3−δ_ (x = 0.1, 0.2) were prepared by pressing the powder in dies and sintering in air at 950 °C for 12 h; finally, the pellet was placed in a tube furnace with 5% H_2_/95% N_2_ flow for 15 h at 900 °C. The densities of the pellets were around 70%–75% of the crystallographic value, calculated from the mass and geometrical volume. Thermal expansion of the sintered samples was carried out in a dilatometer Linseis L75/H, between 100 and 900 °C in H_2_(5%)/N_2_(95%). The conductivity was measured between 25 and 850 °C in H_2_(5%)/N_2_(95%), by the four-point method in bar-shaped pellets under DC currents of 100 mA. The currents were applied and collected with a Potenciostat-Galvanostat AUTOLAB PGSTAT 302, ECO CHEMIE.

Single-cell tests were made on electrolyte-supported cells with La_0.8_Sr_0.2_Ga_0.83_Mg_0.17_O_3−δ_ (LSGM) as the electrolyte, SrCo_0.8_Fe_0.2_O_3−δ_ (SCFO) as the cathode material and SrMo_1__−__x_Mg_x_O_3−δ_ (SMMO) as anode material. The LSGM pellets of 20 mm in diameter were sintered at 1450 °C for 20 h and then polished with a diamond wheel to a thickness of 300 µm. La_0.4_Ce_0.6_O_2__−δ_ (LDC) was used as a buffer layer between the anode and the electrolyte in order to prevent the interdiffusion of ionic species between perovskite and electrolyte. Inks of LDC, SMMO and SCFO were prepared with a binder (V-006 from Heraeus, Hanau, Germany). LDC ink was screen-printed onto one side of the LSGM disk followed by a thermal treatment at 1300 °C in air for 1 h. SMMO was subsequently screen printed onto the LDC layer and fired at 1100 °C in air for 1 h. SCFO was finally screen printed onto the other side of the disk and fired at 1050 °C in air for 1 h. The thickness of the anode and cathode was 10 µm. The working electrode area of the cell for both the anode and cathode was 0.25 cm^2^ (0.5 cm × 0.5 cm). Pt gauze with a small amount of Pt paste in separate dots was used as the current collector at both the anodic and the cathodic sides for ensuring electrical contact. The cells were tested in a vertical tubular furnace at 800 and 850 °C; the anode side was fed with pure H_2_, with a flow of 20 mL·min^−^^1^, whereas the cathode worked in air. The fuel-cell tests were performed with an AUTOLAB 302N Potentiostat/Galvanostat by changing the voltage of the cell from 1.2–0.1 V, with steps of 0.010 V, holding 10 s at each step. Current density was calculated by the recorded current flux through the effective area of the cell (0.25 cm^2^). Each VI (voltage-intensity) scan corresponds to one cycle; the activation of the cell was followed in subsequent cycles until the full power of the single cell was reached.

## 3. Result and Discussion

### 3.1. Crystallographic Characterization 

**Figure 1:**
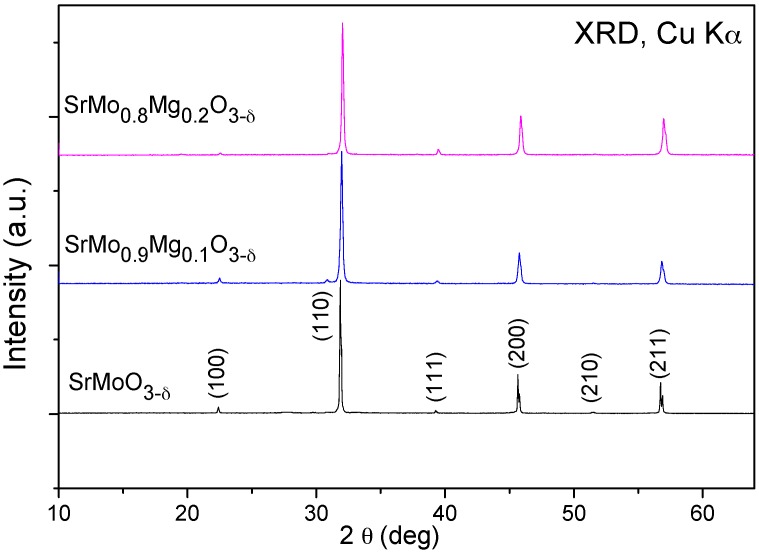
XRD patterns with CuK_α_ radiation for SrMoO_3_ and SrMo_1__−__x_Mg_x_O_3−δ_ (x = 0.1 and 0.2), indexed in a simple cubic perovskite unit cell with a_0_ ≈ 3.95 Å.

The initial characterization of the products was carried out by XRD. SrMo_1__−__x_Mg_x_O_3−δ_ (x = 0.1, 0.2) compounds were obtained as well-crystallized powders. The SrMoO_3_ phase was also prepared as a reference. Figure 1 shows the XRD patterns of the SrMo_1−x_Mg_x_O_3−δ_ (x = 0, 0.1 and 0.2) oxides. The XRD diagrams are characteristic of a cubic perovskite structure with the *Pm-3m* group. The unit-cell parameters obtained for x = 0, 0.1 and 0.2 are 3.9760(3), 3.9739(4) and 3.9654(2) Å, respectively. No impurity phases were detected in any samples.

In order to perform a more comprehensive structural study for the SrMo_1−x_Mg_x_O_3−δ_ (x = 0.1 and 0.2) series, an investigation by NPD at room temperature (RT) for the SrMo_1−x_Mg_x_O_3−δ_ family and high temperature (up to 800 °C) for SrMo_0.9_Mg_0.1_O_3−δ_ was carried out. The structures were refined in the *Pm-3m* group (No. 221), with Z = 1. Sr atoms are located at the 1*b* (½, ½, ½) position; Mo and Mg atoms are randomly distributed at 1*a* (0, 0, 0) sites; and the O oxygen atoms are placed at the 3*d* (½, 0, 0) position. A small oxygen deficiency was observed at room temperature after refining the occupancy factors of the oxygen atoms.

**Figure 2:**
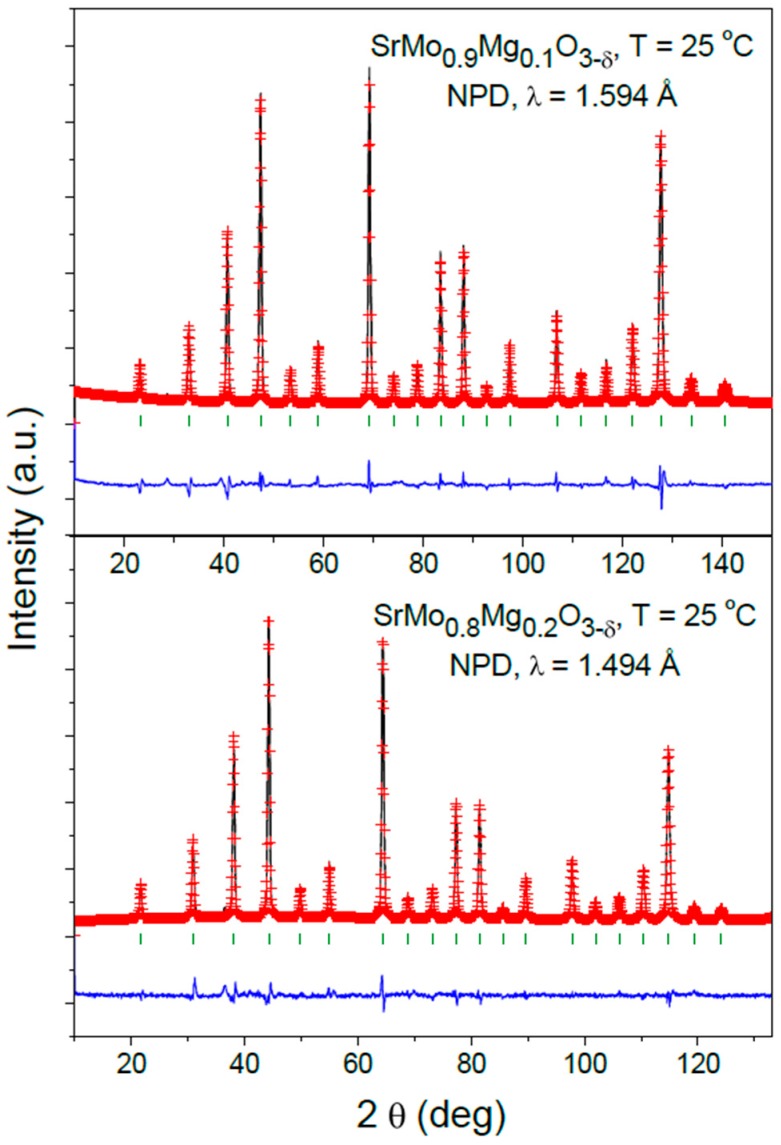
Observed (crosses), calculated (full line) and difference (at the bottom) NPD profiles for SrMo_0.9_Mg_0.1_O_3__−δ_ and SrMo_0.8_Mg_0.2_O_3__−δ_ at 25 °C in air, refined in the cubic *Pm-3m* space group. The vertical markers correspond to the allowed Bragg reflections.

After the complete refinement of the SrMo_1−x_Mg_x_O_3−δ_ (x = 0.1 and 0.2) crystal structures, a good agreement between the observed and calculated NPD patterns at room temperature is shown in Figure 2. Table 1 lists the unit-cell, atomic positions, occupancies, displacement parameters, discrepancy factors and interatomic distances after the Rietveld refinements of doped samples at room temperature.

The unit-cell parameters decrease as the amount of Mg in the sample increases. The (Mo,Mg)-O1 bond lengths at room temperature decrease accordingly with Mg-doping from 1.98814(1) Å for the undoped sample to 1.98247(3) Å for the sample with x = 0.2. This happens even though the ionic size of Mg^2+^ (0.72 Å) is higher than Mo^4+^ (0.65 Å) [8]. This fact may suggest that a unit-cell contraction is happening because oxygen vacancies are being created when Mo is partially replaced by Mg, but it is more probable that this cell contraction is related to a partial oxidation of Mo ions (hole doping effect) as Mg^2+^ is introduced into the perovskite, resulting in a mixed-valence state Mo^4+^-Mo^5+^ proportional to the doping rate. There are well-known Mo-containing double perovskites (e.g., Sr_2_FeMoO_6_) reported to have Mo^5+^ions, exhibiting Mo^5+^-Mo^6+^ mixed valence [12]. Similar unit-cell contraction was observed in previous studies of SrMoO_3_ doped with 10%, 20% and 30% Fe, where the ionic size of high-spin Fe^3+^ is practically the same as Mo^4+^, and the cell is considerably shrunken [6] at room temperature. On the other hand, the oxygen occupancy also evolves with Mg^2+^ doping, being slightly deficient for x = 0.1 (2.985(3) O per formula unit) and significantly more deficient for x = 0.2 (2.856(3) per formula unit) at room temperature.

**Figure 3:**
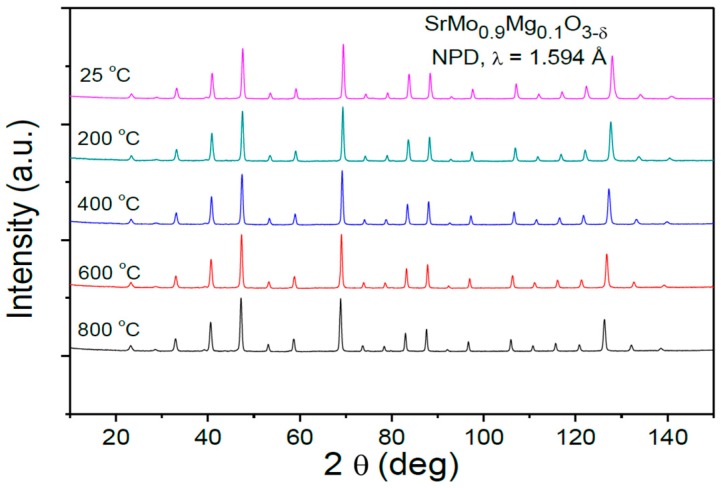
Thermal evolution of the NPD patterns for SrMo_0.9_Mg_0.1_O_3−δ_ between RT and 800 °C.

The thermal evolution of the crystal structure under the anode conditions of an SOFC was studied by NPD for the x = 0.1 oxide. The NPD patterns are illustrated in Figure 3. No structural transitions in the temperature range under study (25–800 °C) were found. 

**Figure 4:**
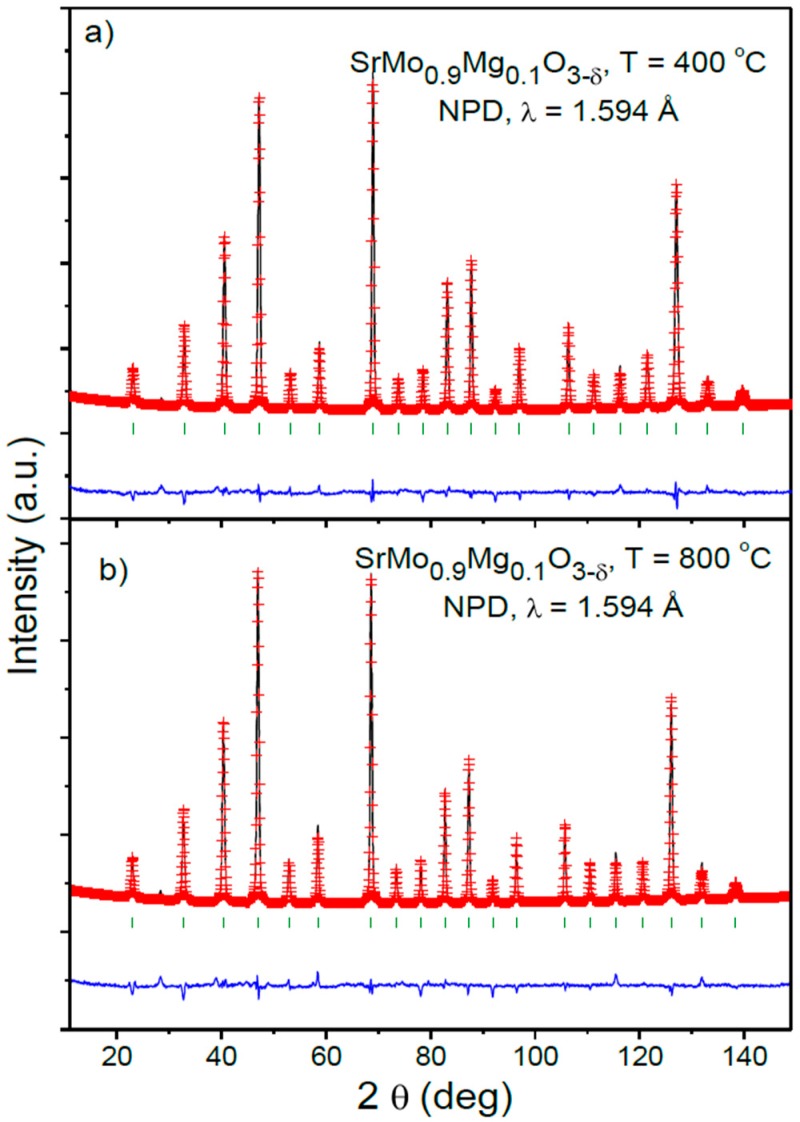
Observed (crosses), calculated (full line) and difference (at the bottom) NPD profiles for SrMo_0.9_Mg_0.1_O_3__−δ_ at (**a**) 400 and (**b**) 800 °C in vacuum (PO_2_ = 10^−^^6^ Torr), refined in the cubic *Pm-3m* space group. The vertical markers correspond to the allowed Bragg reflections.

Figure 4 illustrates the good agreement between the observed and calculated NPD patterns for the sample with x = 0.1 at 400 and 800 °C. Table 2 includes the structural parameters after the refinement of the SrMo_0.9_Mg_0.1_O_3−δ_ structure at the different temperatures under study.

**Figure 5:**
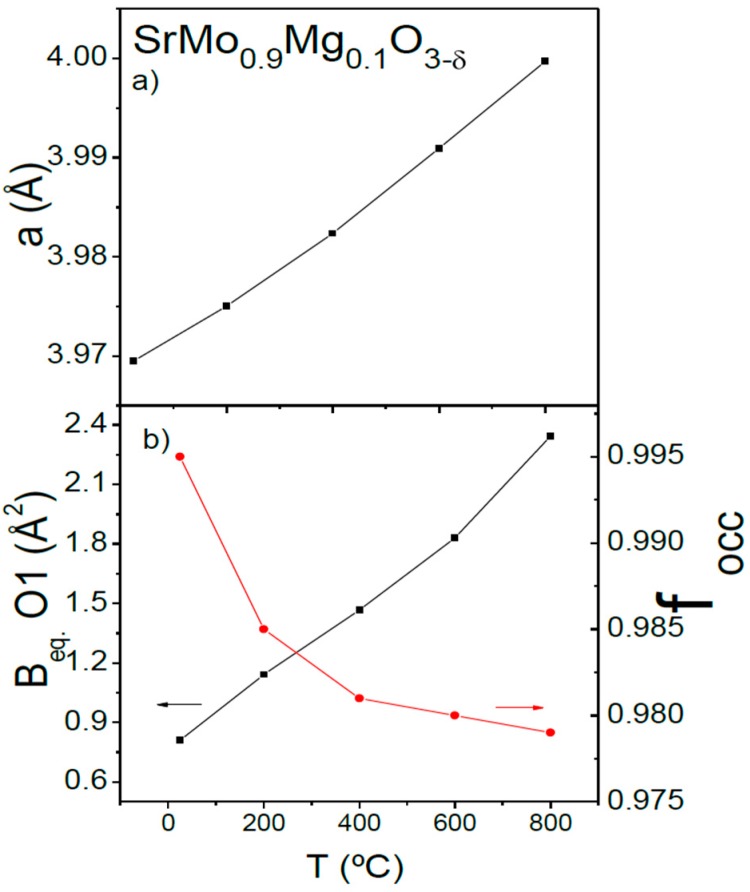
Thermal variation of (**a**) the unit-cell parameter and (**b**) the equivalent isotropic displacement factor for O atoms (left axis) and the oxygen occupancy factor (right axis), from in situ NPD data.

Figure 5a shows the temperature variation of the unit-cell parameters for SrMo_0.9_Mg_0.1_O_3−δ_. The unit-cell parameters monotonically increase when heating the sample due to the expansion of the chemical bonds. The thermal evolution of the oxygen content in air was also studied by neutron diffraction. Figure 5b (right axis) illustrates the temperature variation of the oxygen vacancies concentration for SrMo_0.9_Mg_0.1_O_3−δ_. The oxygen content decreases when heating the sample in vacuum from SrMo_0.9_Mg_0.1_O_2.985(3)_ for x = 0.1, almost stoichiometric at room temperature, to SrMo_0.9_Mg_0.1_O_2.937(3)_ at 800 °C. As the sample is heated, the mixed-valence Mo^4+^-Mo^5+^ is reduced to Mo^4+^, generating oxygen vacancies. Figure 5b (left axis) shows the equivalent isotropic displacement factors of oxygen atoms (B_eq_) increasing from 0.81 at 25 °C to 2.34 Å^2^ at 800 °C. This feature, along with the presence of oxygen vacancies, indicates a high mobility of these atoms, allowing the required O^2−^ motion across the three-dimensional network and providing the material with a good ionic conductivity at the working temperatures of an SOFC.

**Figure 6:**
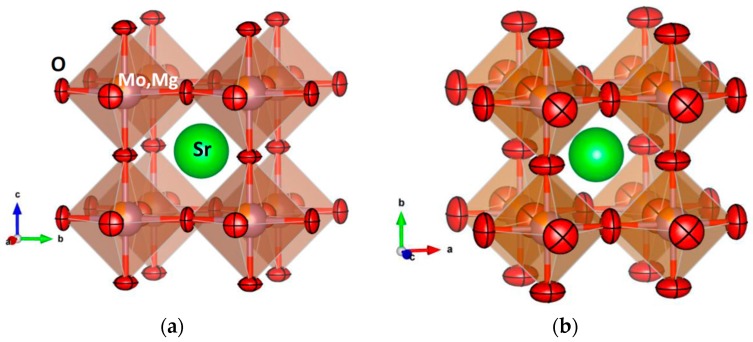
View of the crystal structure of the SrMo_1−x_Mg_x_O_3−δ_ oxides, defined in a simple-cubic, primitive unit cell, showing the evolution of the thermal ellipsoids for oxygen atoms between (**a**) 200 °C and (**b**) 800 °C.

For the cations (Sr, Mo, Mg), the thermal displacement parameters are constrained to be spherical. For O, the anisotropy of the thermal ellipsoids is patent, with the smallest thermal motions along the (Mo,Mg)-O bonds. The magnitude of the thermal motions is monotonically enhanced with temperature, as shown in Table 1. In the entire temperature regime, the O oblate ellipsoids, flattened along the Mo-O-Mo directions, are orientated along the [001] directions. Figure 6 shows the crystal structure of SrMo_1−x_Mg_x_O_3−__δ_ highlighting the evolution of the anisotropic displacements between 200 and 800 °C, with 95% probability for the O nuclear density. At 800 °C, the root mean square (r.m.s.) displacements of O are 0.194 Å perpendicular to the Mo-Mo distance and 0.117 Å parallel to it. The disk-shaped ellipsoids are the result of the strong covalent bonding between Mo^4+^-Mo^5+^ and O; SrMoO_3_ is well known to exhibit band conduction properties by virtue of the robust covalent mixing between 4*d* Mo orbitals and O 2*p* oxygen orbitals, strongly overlapping across 180° Mo-O-Mo angles. Such strong chemical bonds impede the thermal motion along the bonds, in such a way that O atoms exhibit degrees of freedom in the plane perpendicular to the bonding direction. This is in contrast with the prolate ellipsoids observed in other MIEC oxides, like Ba_0.9_Co_0.7_Fe_0.2_Nb_0.1_O_3−δ_ [13], which suggests a breathing of the (Co,Fe,Nb)O_6_ octahedra upon the migration of the oxygen vacancies across the solid. In that case, the average (Co,Fe) oxidation state varies between 2.84+ and 2.02+ in the 25–800 °C temperature range, thus involving much less covalent chemical bonds within the perovskite octahedra, which make possible the less-frequent prolate kind of thermal ellipsoids.

### 3.2. Thermal Analysis

**Figure 7:**
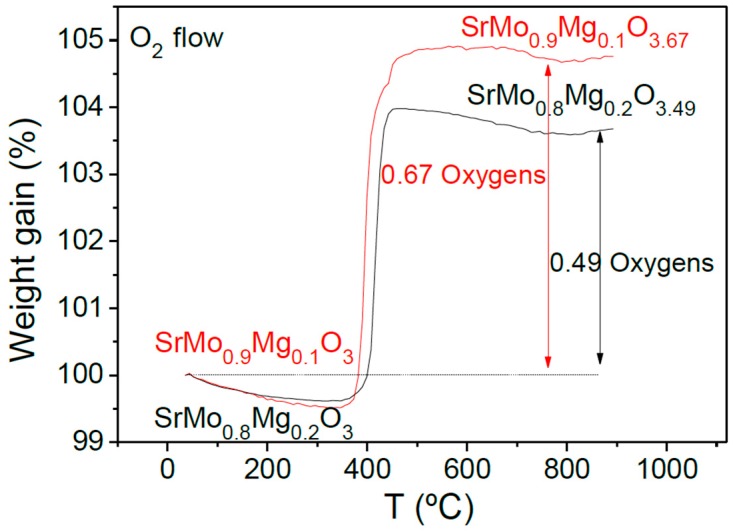
Thermal analysis in O_2_ flow (TG curve) of SrMo_0.9_Mg_0.1_O_3__−δ_ and SrMo_0.8_Mg_0.2_O_3__−δ_ perovskites, showing an oxidation step to a scheelite phase.

The oxidation of the samples by incorporation of oxygen was followed by thermogravimetric analysis carried out in O_2_ flow from 35–900 °C. Figure 7 shows the TGA curves for the SrMo_1−x_Mg_x_O_3−δ_ (x = 0.1 and 0.2) samples. The curves indicate an incorporation of 0.67 oxygen atoms per formula unit for the sample with x = 0.1 and 0.49 oxygens for x = 0.2. As the samples are heated, the oxidation of the perovskite compounds is produced, resulting in crystalline phases with a scheelite-type structure. The incorporation of the oxygen atoms occurs in the 350–500 °C temperature range. The Mo final valence after the oxidation is 5.71+ for x = 0.1 and 5.73+ for x = 0.2.

**Figure 8:**
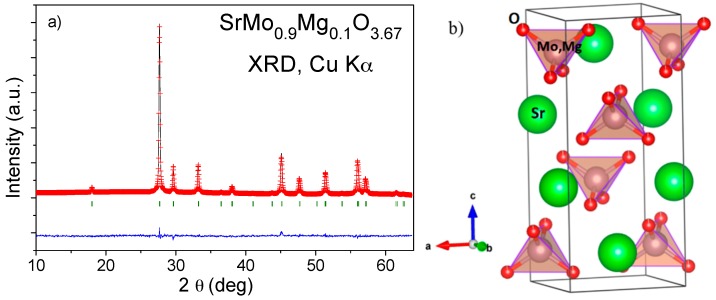
(**a**) Rietveld plot after the structural refinement from XRD data of the oxidation product for SrMo_0.9_Mg_0.1_O_3.67_ scheelite; (**b**) view of the scheelite crystal structure.

Figure 8 shows the refined XRD pattern for the SrMo_0.9_Mg_0.1_O_3.67_ scheelite phase in the space group *I*4_1_*/a* (No. 88) after thermogravimetric analysis in O_2_ flow. Sr atoms are situated at the 4*b* (0, ¼, ⅝) position; Mo and Mg atoms are randomly distributed at 4*a* (0, ¼, ⅛) sites; and O1 oxygen atoms are located at the 16*f* (x, y, z) position. The subsequent heat treatment of the oxidized scheelite phase in reducing (5% H_2_/95% N_2_) atmosphere restored the reduced perovskite phase, confirming the reversibility required in redox cycles. The scheelite structure is a superstructure of fluorite where all of the Mo ions are tetrahedrally coordinated to oxygen atoms, as shown in Figure 8b, with an ordered arrangement of Sr and Mo cations. The tetrahedral units are not connected, whereas the larger Sr cations show eight-fold coordination. A more accurate NPD study would be necessary to determine the oxygen occupancy and interatomic distances to Sr and Mo, in this potentially interesting oxygen-defective scheelite phase.

### 3.3. Thermal Expansion Measurements

**Figure 9:**
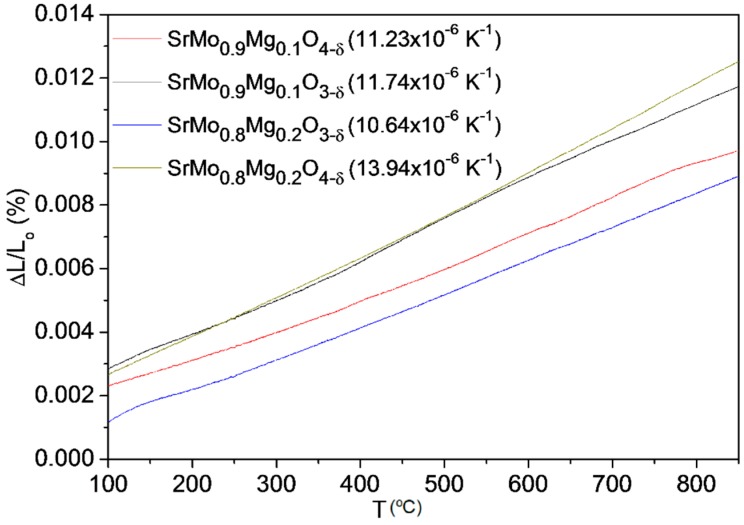
Thermal expansion determined by dilatometry of the SrMo_1__−__x_Mg_x_O_3−δ_ and SrMo_1__−__x_Mg_x_O_4__−δ_ series.

In order to probe the mechanical compatibility of our materials with the other cell components, thermal expansion measurements in dense samples were performed in a 5% H_2_/95% N_2_ atmosphere. The dilatometric analysis was carried out between 25 and 900 °C for several cycles; the data were only recorded during the heating process. Figure 9 shows the thermal expansion for SrMo_1−x_Mg_x_O_3–δ_ (x = 0.1 and 0.2) and SrMo_1−x_Mg_x_O_4–δ_ (x = 0.1 and 0.2). No abrupt changes in the entire temperature measuring range were found. TECs measured in 5% H_2_/95% N_2_ atmosphere for perovskite phases and an air atmosphere for scheelite phases between 400 and 850 °C are included in Figure 9. The TEC value for SrMo_0.9_Mg_0.1_O_3−δ_ is in concordance with that obtained from NPD data in the heating run, of 10.93 × 10^−6^ K^−1^.

The TECs obtained for the perovskite and scheelite phases are reasonably similar and fit with the general SOFC electrolytes values, so no mechanical compatibility problems should be expected during the oxidation-reduction cycles. For the x = 0.1 compound, the TEC coefficients for SrMo_1__−__x_Mg_x_O_3−δ_ and SrMo_1__−__x_Mg_x_O_4__−δ_ are indeed very similar, exhibiting values of 11.74 × 10^−6^ and 11.23 × 10^−6^∙K^−1^, respectively. For x = 0.2, there is a bigger difference (10.64 × 10^−6^ and 13.94 × 10^−6^∙K^−1^, respectively), which could induce a certain redox instability.

### 3.4. Electrical Conductivity Measurements

**Figure 10:**
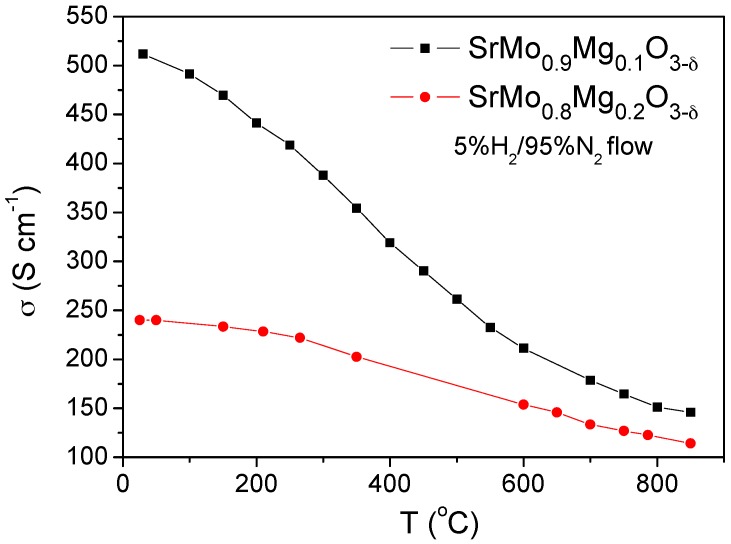
Dc-conductivity as a function of temperature for SrMo_1__−__x_Mg_x_O_3__−δ_ (x = 0.1 and 0.2).

Figure 10 shows the thermal variation of the electrical conductivity of SrMo_1−x_Mg_x_O_3−δ_ (x = 0.1 and 0.2). The resistance was measured by the *dc* four-probe method; a current of 100 mA was applied, and the potential drop was recorded in an Autolab 302N Potentiostat-Galvanostat. Figure 10 illustrates the reduced phases with the perovskite structure featuring a metallic–like conductivity under reducing conditions in both cases. 

Figure 10 illustrates a clear reduction in the electrical conductivity when the Mg content increases, since Mg^2+^ perturbs the conduction paths via Mo-O-Mo chemical bonds, giving total conductivity values at the operating temperature (850 °C) of 146 and 114 S∙cm^−1^ for x = 0.1 and 0.2, respectively. These values are, in any case, sufficiently high for the correct performance of these materials as anodes in SOFC. For instance, σ values of 175 and 160 Scm^−1^ were described for SrMo_0.9_Fe_0.1_O_3−δ_ [6] and SrMo_0.9_Cr_0.1_O_3−δ_ [7] at 850 °C, showing an excellent performance in the hydrogen oxidation reaction in SOFC.

### 3.5. Chemical Compatibility

**Figure 11:**
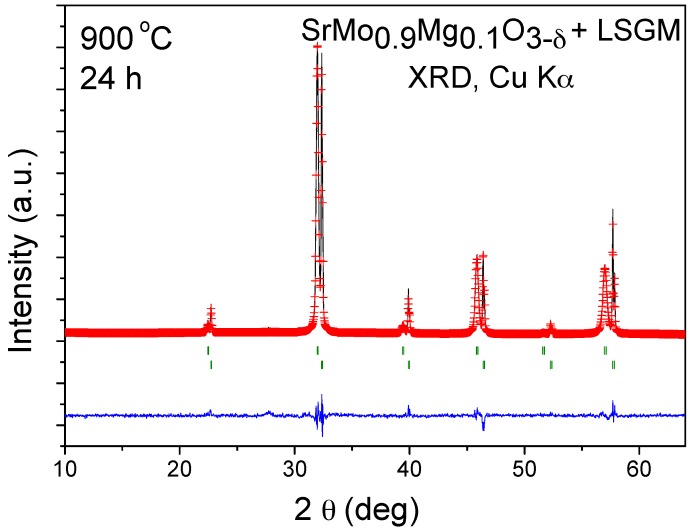
Rietveld-refined XRD profiles of a mixture of LSGM and SrMo_0.9_Mg_0.1_O_3__−δ_ after a thermal treatment at 900 °C in H_2_(5%)/N_2_, showing no reaction products between both phases other than the initial reactants. The first and second series of Bragg positions correspond to LSGM and SrMo_0.9_Mg_0.1_O_3__−δ_, respectively.

The chemical compatibility of SrMo_1−x_Mg_x_O_3−δ_ series with La_0.8_Sr_0.2_Ga_0.83_Mg_0.17_O_3−δ_ (LSGM) electrolyte has been studied by mixing of both powdered samples and heating the mixture at 900 °C under H_2_/N_2_ (5%/95%) atmosphere for 24 h. Figure 11 shows the Rietveld analysis of SrMo_0.9_Mg_0.1_O_3−δ_, consisting of a mixture of both unchanged phases, so no unwanted secondary phases will be formed during the operation in single cells. The same result was obtained for the compound with x = 0.2.

### 3.6. Fuel-Cell Tests

**Figure 12:**
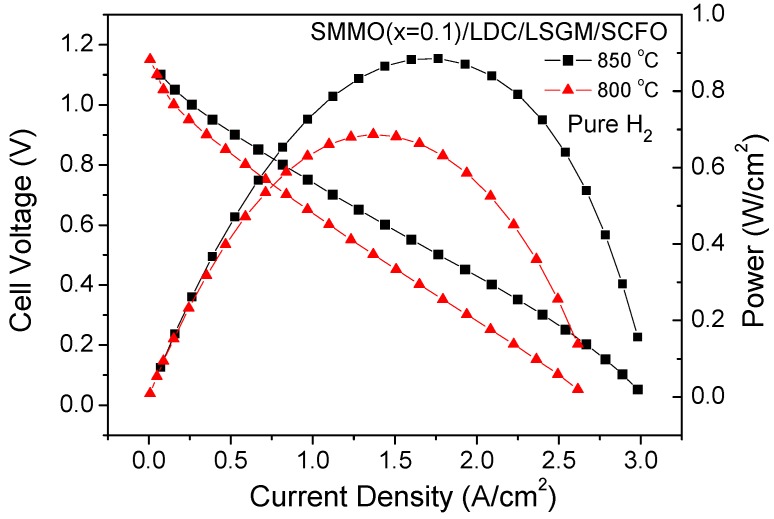
Cell voltage (left axis) and power density (right axis) as a function of the current density for the test cell with the configuration SMMO (x = 0.1)/LDC/LSGM/SCFO in pure H_2_ measured at T = 800 and 850 °C.

In order to study the behavior of SrMo_1−x_Mg_x_O_3−δ_ (x = 0.1 and 0.2) as anodes in solid oxide fuel cells, a single cell for each sample was prepared in an electrolyte-supported configuration using a 300 µm-thick LSGM electrolyte, and the output power was measured at 800 and 850 °C. Figure 12 illustrates the cell voltage and power density as a function of current density at these temperatures for the single cell fed with pure H_2_ for the x = 0.1 anode. The maximum power densities generated by the cell were 684 and 887 mW/cm^2^, respectively. 

**Figure 13:**
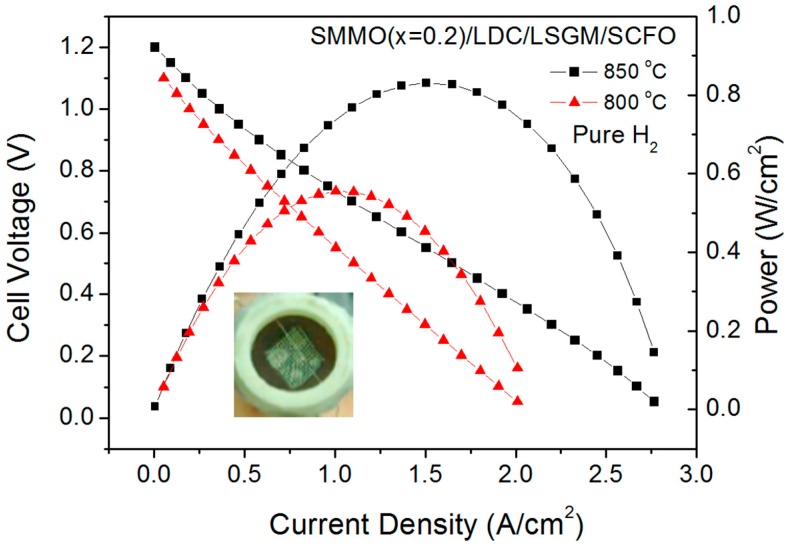
Cell voltage (left axis) and power density (right axis) as a function of the current density for the test cell with the configuration SMMO (x = 0.2)/lanthanum-dope ceria (LDC)/LSGM/SCFO in pure H_2_ measured at T = 800 and 850 °C. The inset shows a view of the cathodic side of the single cell.

Figure 13 shows the cell voltage and power density as a function of current density at the same temperatures for the anode x = 0.2. The maximum power densities generated by the cell were 555 and 832 mW/cm^2^, respectively. The inset of Figure 13 illustrates a view of the cathode side of the cell.

Although both anodes have an exceptional behavior, a slight decrease of the output power of the single cells is observed for x = 0.2 with respect to the x = 0.1 anode. This reduction of the power density could be related to the decrease in the Mo contents of the anode in the x = 0.2 sample, since apparently, molybdenum is responsible for the catalytic oxidation of the fuel, as has been observed in other Mo-containing anodes [6,14]. Additionally, the observed reduction of the electrical conductivity (Figure 10) in the whole range of measured temperatures also contributes to the deterioration of the output power for this anode material. 

In a previous work [7], an additional test using Au gauze with a small amount of Au paste as the current collector instead of Pt gauze was carried out to check if Pt could promote the catalytic process of O_2_ reduction or fuel oxidation as suggested by some authors [15,16,17], increasing the power density and covering up the true activity of the oxides selected as electrodes. In this work, the maximum power densities generated by the cell were even higher than with Pt gauze. Since Au has no catalytic properties, this test implies that the observed activity comes entirely from the anode material. 

In order to compare the performance of our SrMo_1−x_Mg_x_O_3−δ_ (x = 0.1 and 0.2) anodes with other SrMo_1−x_M_x_O_3−δ_ (M = Fe and Cr) anodes, in previous works [6,7], an identical single cell with these anodes was also made and measured. Similar power outputs were observed in these cases (874 mW/cm^2^ for SrMo_0.9_Fe_0.1_O_3−δ_ and 695 mW/cm^2^ for SrMo_0.9_Cr_0.1_O_3−δ_ at 850 °C), demonstrating that our anodes are even slightly better than these materials. Moreover, in the long-term performance, the Mg^2+^-doped anodes are believed to be superior due to the absence of interdiffusion cationic effects, since Mg is also contained in the LSGM electrolyte. 

## 4. Conclusions

In this study, we have shown that SrMo_1−x_Mg_x_O_3−δ_ (x = 0.1 and 0.2) oxides crystallize in a cubic perovskite structure (*Pm-3m*) where a mixed Mo^4+^-Mo^5+^ oxidation state is present at RT; NPD data unveil the creation of an appreciable amount of oxygen vacancies at high temperatures, under the low pO_2_ working conditions of an SOFC. The anisotropic displacements for O atoms, conforming flattened ellipsoids, correspond to the highly covalent Mo-O bonds. SrMo_1−x_Mg_x_O_3−δ_ (x = 0.1 and 0.2) oxides can be successfully used as anode materials in SOFC test cells in an electrolyte-supported configuration using a 300 µm-thick LSGM electrolyte. Excellent maximum output powers of 887 and 832 mW/cm^2^ are obtained for x = 0.1, 0.2, respectively, at 850 °C, using pure H_2_ as a fuel. The sufficiently large number of oxygen vacancies combined with high thermal displacement factors suggest a high ionic conductivity at the operating temperatures, constituting MIEC-type materials together with the high electronic conductivity associated with the pristine SrMoO_3_ sample. In addition, the reversibility of the reduction-oxidation between the Sr(Mo,Mg)O_4__−δ_ scheelite and Sr(Mo,Mg)O_3−δ_ perovskite phases makes possible the required cyclability of the cells. The obtained TECs, ranging between 13.94 × 10^−6^ and 10.64 ×10^−6^ K^−1^, are perfectly compatible with the usual SOFC electrolytes. Finally, excellent chemical compatibility was observed with the electrolyte LSGM for 24 h at 900 °C.

## Figures and Tables

**Table 1:** Unit-cell and thermal parameters for SrMo_1−x_Mg_x_O_3−δ_ (x = 0, 0.1 and 0.2) in the cubic *Pm-3m* (No. 221) space group, from neutron power diffraction (NPD) at RT. Sr is placed at the 1*b* (½, ½, ½), (Mo,Mg) at the 1*a* (0, 0, 0) and O1 at the 3*d* (½, 0, 0) position.

SrMo_1−x_Mg_x_O_3−δ_	x = 0 ^a^	x = 0.1	x = 0.2
a (Å)	3.97629(3)	3.96948(1)	3.96494(6)
V (Å^3^)	62.869(7)	62.546(1)	62.332(2)
Sr 1*b* (½, ½, ½)			
B_iso_ (Å^2^)	0.77(3)	0.815(3)	1.223(3)
f_occ_	1.00	1.00	1.00
Mo/Mg 1*a* (0, 0, 0)			
B_iso_ (Å^2^)	0.55(4)	0.245(3)	0.575(2)
Mo/Mg f_occ_	1.00	0.894(1)/0.108(1)	0.744(1)/0.255(1)
O1 3*d* (½, 0, 0)			
β_11_ *	-	41(7)	103(8)
β_22_ *	-	172(5)	219(5)
β_33_ *	-	172(5)	219(5)
B_eq_ (Å^2^)	0.75(10)	0.81	1.14
f_occ_	1.00	0.995(1)	0.982(1)
Reliability factors			
χ^2^	-	5.35	1.69
R_p_ (%)	-	3.97	4.64
R_wp_ (%)	-	5.17	6.22
R_exp_ (%)	-	2.23	4.76
R_Bragg_ (%)	-	2.84	2.70
Distances (Å)			
(Sr)–(O1)	-	2.80684(3)	2.80364(3)
(Mo/Mg)–(O1)	1.98814(1)	1.98474(2)	1.98247(3)

^a^ Taken from [11]; * anisotropic betas (×10^4^); β_12_ = β_13_ = β_23_ = 0.

**Table 2:** Unit-cell, thermal parameters and selected distances (Å) for SrMo_0.9_Mg_0.1_O_3−δ_ in the cubic *Pm-3m* (No. 221) space group, from NPD from RT (25 °C) to 800 °C.

SrMo_0.9_Mg_0.1_O_3−δ_	25 °C	200 °C	400 °C	600 °C	800 °C
a (Å)	3.96948(1)	3.97503(7)	3.98237(6)	3.99096(6)	3.99971(6)
V (Å)^3^	62.546(1)	62.809(2)	63.158(2)	63.567(2)	63.986(2)
Sr 1*b* (½, ½, ½)					
B_iso_ (Å^2^)	0.815(3)	1.238(3)	1.633(3)	2.024(3)	2.452(4)
f_occ_	1.00	1.00	1.00	1.00	1.00
Mo/Mg 1*a* (0, 0, 0)					
B_iso_ (Å^2^)	0.245(3)	0.3783)	0.465(3)	0.678(3)	0.886(3)
Mo/Mg f_occ_	0.894(1)/0.108(1)	0.894(1)/0.108(1)	0.894(1)/0.108(1)	0.894(1)/0.108(1)	0.894(1)/0.108(1)
O1 3*d* (½, 0, 0)					
β_11_ *	41(7)	81(8)	97(7)	137(8)	170(8)
β_22_ *	172(5)	231(6)	298(5)	381(6)	465(6)
β_33_ *	172(5)	231(6)	298(5)	381(6)	465(6)
B_eq_ (Å^2^)	0.81	1.14	1.47	1.83	2.34
f_occ_	0.995(1)	0.985(3)	0.988(1)	0.980(1)	0.979(1)
Reliability factors					
χ^2^	5.35	2.02	2.79	2.65	2.88
R_p_ (%)	3.97	3.93	3.25	3.19	2.89
R_wp_ (%)	5.17	5.01	4.19	4.12	3.82
R_exp_ (%)	2.23	3.53	2.51	2.53	2.25
R_Bragg_ (%)	2.84	2.69	3.97	3.96	3.99
Distances (Å)					
(Sr)-(O1)	2.80684(3)	2.81077(4)	2.81596(3)	2.82204(3)	2.82822(3)
(Mo/Mg)-(O1)	1.98474(2)	1.98752(4)	1.99119(3)	1.99548(3)	1.99986(3)

* Anisotropic betas (×10^4^); β_12_ = β_13_ = β_23_ = 0.
